# Comparative Identification of MicroRNAs in *Apis cerana cerana* Workers’ Midguts in Response to *Nosema ceranae* Invasion

**DOI:** 10.3390/insects10090258

**Published:** 2019-08-21

**Authors:** Dafu Chen, Yu Du, Huazhi Chen, Yuanchan Fan, Xiaoxue Fan, Zhiwei Zhu, Jie Wang, Cuiling Xiong, Yanzhen Zheng, Chunsheng Hou, Qingyun Diao, Rui Guo

**Affiliations:** 1 College of Bee Science, Fujian Agriculture and Forestry University, Fuzhou 350002, China; 2 Institute of Apicultural Research, Chinese Academy of Agricultural Sciences, Beijing 100093, China

**Keywords:** *Apis cerana cerana*, midgut, immune defense, *Nosema ceranae*, microRNA, target mRNA, regulatory network

## Abstract

Here, the expression profiles and differentially expressed miRNAs (DEmiRNAs) in the midguts of *Apis cerana cerana* workers at 7 d and 10 d post-inoculation (dpi) with *N. ceranae* were investigated via small RNA sequencing and bioinformatics. Five hundred and twenty nine (529) known miRNAs and 25 novel miRNAs were identified in this study, and the expression of 16 predicted miRNAs was confirmed by Stem-loop RT-PCR. A total of 14 DEmiRNAs were detected in the midgut at 7 dpi, including eight up-regulated and six down-regulated miRNAs, while 12 DEmiRNAs were observed in the midgut at 10 dpi, including nine up-regulated and three down-regulated ones. Additionally, five DEmiRNAs were shared, while nine and seven DEmiRNAs were specifically expressed in midguts at 7 dpi and 10 dpi. Gene ontology analysis suggested some DEmiRNAs and corresponding target mRNAs were involved in various functions including immune system processes and response to stimulus. KEGG pathway analysis shed light on the potential functions of some DEmiRNAs in regulating target mRNAs engaged in material and energy metabolisms, cellular immunity and the humoral immune system. Further investigation demonstrated a complex regulation network between DEmiRNAs and their target mRNAs, with miR-598-y, miR-252-y, miR-92-x and miR-3654-y at the center. Our results can facilitate future exploration of the regulatory roles of miRNAs in host responses to *N. ceranae*, and provide potential candidates for further investigation of the molecular mechanisms underlying eastern honeybee-microsporidian interactions.

## 1. Introduction

Honeybees play vital roles not only in the pollination of crops and wild flora, but also in the support of critical ecosystem balance [1,2]. In addition, honeybees serve as key models for studying development, social behavior, and disease transmission [3,4]. Western honeybees (*Apis mellifera*) and eastern honeybees (*Apis cerana*), the two best-known honeybee species, have been used for honey production and crop pollination. Compared with *A. mellifera*, *A. cerana* has some prominent advantages, such as long flight duration, adaptation to extreme weather conditions, frequent grooming and hygienic behaviors, and cooperative group-level defenses [5]. *Apis cerana cerana*, a subspecies of *A. cerana*, is an important endemic species that is crucial to ecosystem balance and environmental improvement in China [6].

Nosemosis is a serious disease in adult honeybees caused by several *Nosema* species. *Nosema* infection occurs through the ingestion of spores in contaminated food or water, followed by the germination of these spores triggered by the physical and chemical conditions of the midgut. The genetic material is then transferred into the host cell of the midgut epithelium where it multiplies, and finally, the new spores are excreted from the bee in the feces, which provides new sources of the infection via cleaning and feeding activities in the colonies, or are disseminated into the environment [7,8,9]. Microsporidia are spore-forming and obligate intracellular fungal pathogens, which can infect a wide variety of hosts, ranging from insects to mammals [10]. *N. ceranae* is a widespread microsporidian pathogen of honeybees, which was first discovered and described from *A. cerana* near Beijing, China [11], and shortly afterwards, it was reported to have infected *A. mellifera* in Europe and Taiwan province [12,13]. At present, *N. ceranae* has been identified in colonies of *A. mellifera* all over the world [14,15] *N. ceranae* is infective to all castes in the colony, including queens, drones and workers [9]. It can remarkably reduce colony strength and productivity but also interacts with other environmental stressors to weaken colony health [16,17].

MicroRNAs (miRNAs) are single-stranded, highly conserved, non-coding RNA (ncRNA) molecules of 19–24 nucleotides (nt) in length that negatively regulate gene expression by targeting specific sites in the 3′ untranslated region (UTR) of mRNAs at the post transcriptional level or by mediating the degradation of the target mRNAs [18]. Recent evidence demonstrated miRNAs can also positively regulate gene expression in metazoan and exert regulatory function via binding to CDS or 5′ UTR of mRNAs [19,20,21]. Studies over the past years have shown that miRNAs are able to play key parts in diverse biological processes, including cell proliferation [22], apoptosis [23], morphogenesis [24], and metabolism [25], as well as playing roles in stress [26] and immune responses [27].

In comparison to other insects such as *Drosophila* and the silkworm, little research has been done on the roles of miRNAs during the host-parasite interactions in honeybees. Huang and colleagues performed deep sequencing of western honeybee miRNAs daily across the six day reproductive cycle of *N. ceranae* and detected 17 DEmiRNAs that target more than 400 mRNAs involved in several pathways including metabolism [28]. In eastern honeybees infected with *N. ceranae*, a series of changes are accompanied by alterations in gene expression, and miRNAs are likely to be involved in this process. However, there has been no report of studies addressing this issue of *A. c. cerana* until now. In the current work, in order to investigate eastern honeybee miRNAs and expand upon novel insights into host-parasite interactions in *A. c. cerana*, we utilized deep sequencing and bioinformatics to analyze the dynamic miRNA expression profiles and differentially expressed miRNAs (DEmiRNAs) of *A. c. cerana* after *N. ceranae* invasion and identified the corresponding target mRNAs and pathways involved. Our results provide novel insights into the host-pathogen interactions between *A. c. cerana* and *N. ceranae* and facilitate further investigation of miRNA function in eastern honeybee responses to *N. ceranae*.

## 2. Materials and Methods

### 2.1. Preparation of N. ceranae Spores

Foraging bees were collected from a heavily infected colony in an apiary of *A. c. cerana* in Fuzhou city, Fujian Province, China. The infected honeybees were kept at −20 °C for 20 min to anesthetize them, followed by purification of fresh spores of *N. ceranae* [29,30] with some modifications. In brief, the midguts of infected workers were removed and crushed in sterile water and filtered with four layers of gauze to remove tissue debris. Following centrifugation of the filtered suspension at 6000× g for 5 min, the supernatant was discarded, and the resuspended pellet was further purified on a discontinuous Percoll gradient (Solarbio, Beijing, China) containing 5 mL each of 25%, 50%, 75% and 100% Percoll solution. The spore suspension was overlaid onto the gradient and centrifuged at 18,000× *g* for 90 min at 4 °C. The spore pellet was carefully extracted with a syringe and then centrifuged again on a discontinuous Percoll gradient to obtain clean spores (Figure S1A), which were immediately used for experimental infection of *A. c. cerana* workers.

### 2.2. Experimental Inoculation of Honeybees

Frames of a sealed brood comb from a healthy colony of *A. c. cerana* (*Nosema*-free, as verified by PCR) kept in an experimental apiary of the College of Bee Science, Fujian Agriculture and Forestry University, were swiftly transferred to the laboratory, and kept in an incubator at 34 ± 2 °C to provide newly emerged *Nosema*-free workers. Workers were carefully removed, confined in plastic cages in groups of 30, and kept in an incubator at 34 ± 2 °C. According to the standard method previously described [31], 24 h after eclosion, the workers were starved for 2 h and then each was fed with 5 μL of a 50% sucrose (*w*/*w* in water) solution containing 1 × 10^6^ *N. ceranae* spores [32], as shown in Figure S1B. The presence of *N. ceranae* spores confirmed by 5s rRNA gene (EF091879.1) of *N. ceranae* and the absence of *Nosema apis* spores were confirmed by PCR (Figure S1C, Table S1) using previously described primers [15]. To exclude the interference from several common bee viruses including acute bee paralysis virus (ABPV), black queen cell virus (BQCV), deformed wing virus (DWV), chronic bee paralysis virus (CBPV), kashmir bee virus (KBV), israeli acute paralysis virus (IAPV) and sacbrood virus (SBV), RT-PCR was performed using previously described specific primers (Table S1) [33,34,35,36,37] and total RNA from sucrose solution containing *N. ceranae* spores. Expectedly, agarose gel electrophoresis suggested that only a signal fragment (about 76 bp) was amplified using *N. ceranae* specific primers, while no bands were amplified using specific primers for *N. apis* and several bee viruses (Figure S1C). For *N. ceranae*-treated groups, honeybee workers were held with their mouthparts touching a droplet with the spore solution at the tip of a micropipette until the worker had consumed the entire droplet; workers that did not consume the entire droplet were discarded. For the control group, each worker in three replicates was fed 5 μL of a 50% sucrose solution without *N. ceranae* spores. The honeybees were fed ad libitum with a solution of sucrose (50% *w*/*w*), and the feeders were replaced daily throughout the whole experiment. Each cage was examined daily, and dead bees were removed and counted. Nine bees from each cage in the *N. ceranae*-treated and control groups were collected at 7 d and 10 d post-inoculation (dpi) and then sacrificed, followed by dissection of the intestinal tract and separation of the midgut from the rectum and Malpighian tubules. For each cage, midgut tissues were immediately pooled from all nine workers, frozen in liquid nitrogen and stored at −80 °C until sRNA-seq, Stem-loop RT-PCR and real time quantitative PCR (RT-qPCR) was performed.

### 2.3. Small RNA Isolation, cDNA Library Construction and Deep Sequencing

Firstly, total RNA of each midgut sample in *N. ceranae*-treated and control groups were extracted using TRIzol Reagent (Invitrogen, Carlsbad, CA, USA) following the manufacturer’s protocols. Secondly, DNA contaminants were removed with RNase-freeDNase I (TaKaRa, Beijing, China). The purified RNA quantity and quality were checked using Nanodrop 2000 spectrophotometer (Thermo Fisher, Waltham, MA, USA), and the integrity of the RNA samples were evaluated using Agilent 2100 bioanalyzer (Agilent Technologies, Santa Clara, CA, USA) and only values of 28S/18S ≥ 0.7 and RIN ≥ 7.0 were considered qualified for the subsequent small RNA library construction. Thirdly, RNA molecules in the size range of 18–30 nt were enriched by agarose gel electrophoresis (AGE) and then ligated with 3′ and 5′ RNA adaptors, and fragments with adaptors on both ends were enriched by PCR after reverse transcription. Fourthly, the subsequent cDNAs were purified and enriched by 3.5% AGE to isolate the expected size (140–160 bp) fractions and eliminate unincorporated primers, primer dimer products, and dimerized adaptors. Ultimately, the 12 cDNA libraries were sequenced on Illumina sequencing platform (HiSeq^TM^ 4000) using the single-end technology by GENE DENOVO Biotechnology Co. (Guangzhou, China). The libraries were as follows: AcCK1-1, AcCK1-2 and AcCK1-3 as replicate libraries for normal midguts of workers at 7 dpi with sucrose solution; AcT1-1, AcT1-2 and AcT1-3 as replicate libraries for midguts of workers at 7 dpi with sucrose solution containing *N. ceranae* spores; AcCK2-1, AcCK2-2 and AcCK2-3 as replicate libraries for normal midguts of workers at 10 dpi with sucrose solution; AcT2-1, AcT2-2 and AcT2-3 as replicate libraries for midguts of workers at 10 dpi with sucrose solution containing *N. ceranae* spores.

### 2.4. Quality Control and Sequencing Data Analysis

The raw data were pre-processed to exclude low-quality reads (length < 20 nt and ambiguous N), 5′ adapter, 3′ adapter and poly(A) sequences to gain clean reads, which were aligned against NCBI GeneBank and Rfam databases to remove ncRNA including rRNA, scRNA, snoRNA, snRNA and tRNA. Then, the obtained sequences were compared with exons and introns in the *A. cerana* genome (assembly ACSNU-2.0) to classify mRNA degradation products and the repeat associate miRNA sequences. All the downstream analyses were based on clean reads with high quality.

Currently, only the miRNA information of western honeybee was recorded in miRBase database, while the related information of eastern honeybees, including *A. c. cerana*, was not included. By utilizing Bowtie (v1.1.0) [38], the filtered sequences were analyzed with BLAST search against miRBase 21.0 by allowing at most two mismatches outside of the seed region [39], and small RNAs that matched exist miRNAs of other animal species in miRBase were identified as known miRNAs. The sequences that did not match known miRNAs were used to identify potentially novel miRNA candidates using RNAfold software [40]. Only sequences with typical Stem-loop hairpins and free energy lower than –20 kcal/mol were considered as potential novel miRNAs. The suffixes “-x” and “-y” respectively mean a certain miRNA deriving from the processing of the 5′ and 3′ arms of its precursor, while the suffix “-z” means a certain miRNA with unknown processing direction. Size distribution and saturation analysis were performed using sequencing libraries after all annotation steps. Hierarchical clustering of novel miRNA expression was conducted using heatmap tool in OmicShare (http://www.omicshare.com/tools/).

### 2.5. Analysis of DEmiRNAs

The miRNA expression levels in each sample were normalized to the total number of sequence tags per million (TPM) following formula: normalized expression = mapped read count/total reads×10^6^. Differential expression analysis of two samples was performed using the DEGseq R package [41] and *p* values were adjusted using *q* value. The criteria of *p* value (FDR) < 0.05 and |log_2_(Fold change)| > 1 were set as the threshold for statistically significant differential expression. A positive value indicated upregulation of a miRNA, while a negative value indicated down-regulation.

### 2.6. Prediction of the Target mRNAs of DEmiRNAs and Construction of miRNA-mRNA Regulation Networks

Target mRNAs for miRNAs were predicted with miRanda (v3.3a) [42], RNAhybrid (v2.1.2) +svm_light (v6.01) [43] and TargetFinder (Version: 7.0) [44] software. The input files were miRNA FASTA sequences files. Intersections of the results from the three above mentioned programs comprised the final predicted gene targets. Then, miRNA-mRNA regulation networks were visualized using Cytoscape v.3.2.1 software [45], following parameters: the free energy of the target site/miRNA duplex needs to be lower than −35 kcal/mol and *p* < 0.05.

### 2.7. GO and KEGG Pathway Analyses of DEmiRNA Target mRNAs

To determine their main biological functions, DEmiRNA target mRNAs were annotated by terms in the Gene Ontology (GO) database (http://www.geneontology.org/) using Blast2GO [46], following their numeric orders in the NCBI nr database. Subsequently, GO functional categorizations for all target mRNAs were determined with WEGO software [47], and GO enrichment analysis of functional significance terms in the GO database was performed using the hypergeometric test to identify significantly enriched GO terms for the target mRNAs compared to the genome background. KEGG pathway analyses of the predicted target mRNAs were performed using the KEGG pathway database (http://www.genome.jp/kegg/pathway.html) [48].

### 2.8. Stem-Loop RT-PCR Confirmation of miRNAs

Total RNAs from AcCK1 and AcCK2 samples were isolated using RNAiso plus kit (TaKaRa) and then treated with DNase I (TaKaRa) to remove remaining DNA. According to the previously described method [49], Stem-loop primers, specific forward primers and universal reverse primers (presented in Table S2) were designed using DNAMAN software on basis of the sequences of the randomly selected nine *A. c. cerana* miRNAs, including miR-1943-x, miR-3793-x, miR-252-y, miR-3963-x, miR-8516-x, miR-149-y, miR-7311-y, miR-9008-x, novel-m0014-3p. The primers were synthesized by Sangon Biotech Co., Ltd. (Shanghai, China). One microgram of total RNA was reverse transcribed to cDNA using RevertAid First Strand cDNA Synthesis Kit (TaKaRa) and Stem-loop primers (Table S2). The PCR amplification of randomly selected miRNAs was conducted on a T100 thermo cycler (BIO-RAD) using Premix (TaKaRa) under the following conditions: pre-denaturation step at 94 °C for 5 min; 30 amplification cycles of denaturation at 94 °C for 50 s, annealing at 55 °C for 30 s, and elongation at 72 °C for 1 min; followed by a final elongation step at 72 °C for 10 min. The PCR products were detected on 2% AGE with Genecolor (Gene-Bio, Shenzhen, China) staining.

### 2.9. RT-qPCR Validation of DEmiRNAs

Four DEmiRNAs (miR-6547-x, miR-7311-x, novel-m0008-3p and miR-2779-y) within AcCK1 vs AcT1, and four DEmiRNAs (miR-3726-x, miR-1788-y, miR-3319-y and miR-1672-x) within AcCK2 vs AcT2 were randomly selected for RT-qPCR validation. Total RNA of two control midgut samples (AcCK1, AcCK2) and two *N. ceranae*-infected midgut samples (AcT1, AcT2) were respectively isolated using RNAiso plus kit (TaKaRa), followed by inverse transcription with corresponding Stem-loop primers using the method mentioned above. The generated cDNA was used for RT-qPCR validation with specific forward primer and universal reverse primer. Stem-loop primers and specific forward primers (presented in Table S2) were designed and synthesized using the aforementioned method. RT-qPCR was carried out in an Applied Biosystems QuantStudio 3 (Thermo Fisher, Waltham, MA, USA) in a 20 μL reaction volume containing 1 μL cDNA, 10 μL SYBR Premix (Vazyme, Nanjing, China), 0.2 μL specific forward primer (20 μM), 0.2 μL reverse primer (20 μM), and 8.6 μl DEPC water. The reaction was performed at 95 °C for 5 min, followed by 45 cycles of 94 °C for 15 s, 60 °C for 15 s, and 72 °C for 15 s. The abundance of miRNAs was normalized relative to that of endogenous control snRNA U6. All reactions were performed in triplicate. The threshold cycle (Ct) was determined using the default threshold settings, and the data were analyzed using 2^–ΔΔCt^ program [50]. The experiment was performed three times using three independent biological samples.

### 2.10. Statistical Data Analysis

All statistical analyses were performed using SPSS software (IBM, Armonk, NY, USA) and GraphPad Prism 6.0 software (GraphPad Software Inc., San Diego, CA, USA). Data were presented as mean ± standard deviation (SD). Statistical analysis was calculated using independent-samples *t*-test and one-way ANOVA. Fisher’s exact test was employed to filter the significant GO terms and KEGG pathways using R software 3.3.1. *p* < 0.05 was considered statistically significant.

## 3. Results

### 3.1. Overview of the Small RNA Library from A. c. cerana Workers’ Guts

To identify the miRNA profiles and DEmiRNAs during the responses of *A. c. cerana* workers’ midguts to *N. ceranae* invasion, 12 small RNA libraries were constructed using Illumina sequencing. In total, 127,523,419 raw reads with an average of ~106,269,512 reads per sample were produced, and after filtering out low-quality sequences, 5′ and 3′ adaptors and reads 18 < nt, 122,104,443 clean reads were obtained for further analysis (Table 1). In addition, the Pearson correlation between every sample in each group was above 0.9619 (Figure S2), suggesting sufficient reproducibility and rationality of sampling. A BLAST run against the NCBI GenBank and RFam databases identified 3,611,375 (36.20%) to 5,054,110 (52.00%) unique small RNAs as rRNA, 101,602 (1.02%) to 221,407 (1.97%) as tRNAs, 5123 (0.05%) to 11,137 (0.11%) as snRNAs, and 176 (0.002%) to 584 (0.005%) as snoRNAs (Table S3).

**Figure 1:**
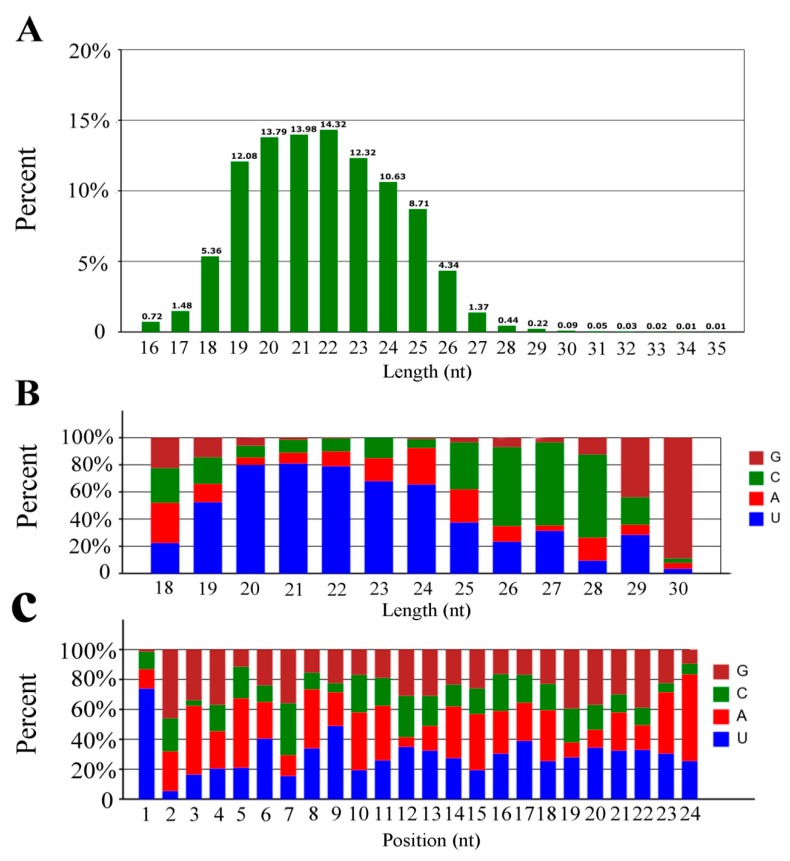
Length distribution and nucleotide bias of the total miRNAs in the untreated and *N. ceranae*-treated *A. c. cerana* workers’ midguts. (**A**) Length distribution; (**B**) First nucleotide bias; (**C**) Nucleotide bias at each position.

Base composition, which is a fundamental feature of miRNA sequences, influences miRNA physiochemical and biochemical properties [51,52]. The sequence lengths of the clean tags were almost all distributed between 16–30 nt, with peaks at 22 nt accounting for 14.32% of all clean reads, followed by 20 nt and 21 nt, which accounted for 13.79% and 13.98% of clean reads, respectively (Figure 1A). The nucleotide bias of miRNAs in the midgut of the *A. c. cerana* worker was further investigated, and the results showed that U was the most common nucleotide at the 5′ end of miRNAs (Figure 1B), which was consistent with other studies that also found U to be the most common base at the extreme 5′ end of miRNAs [53,54,55]. Additionally, analysis of the nucleotide bias at each position showed that U and A were mainly located at the beginning and end of reads of *A. c. cerana* miRNAs (Figure 1C), suggesting that AU base pairing may affect miRNA secondary structure or target recognition [56].

### 3.2. Identification of Known and Novel miRNAs in A. c. cerana Worker’s Midgut

**Figure 2:**
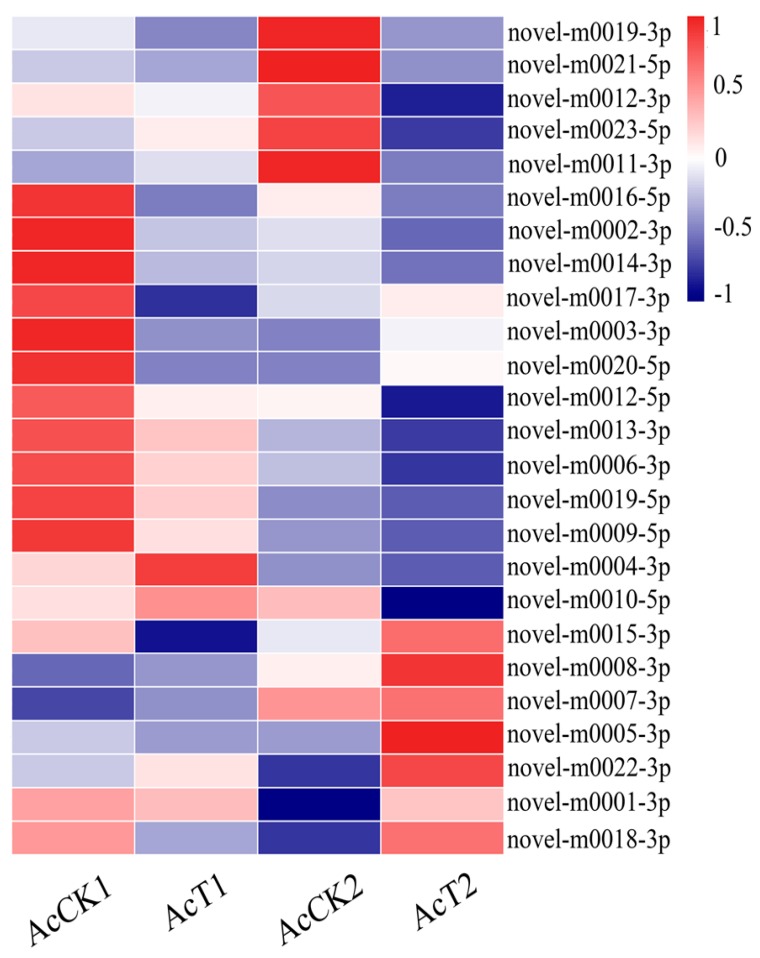
Expression clustering of novel miRNAs in the untreated and *N. ceranae*-treated midguts of *A. c. cerana* workers.

**Figure 3:**
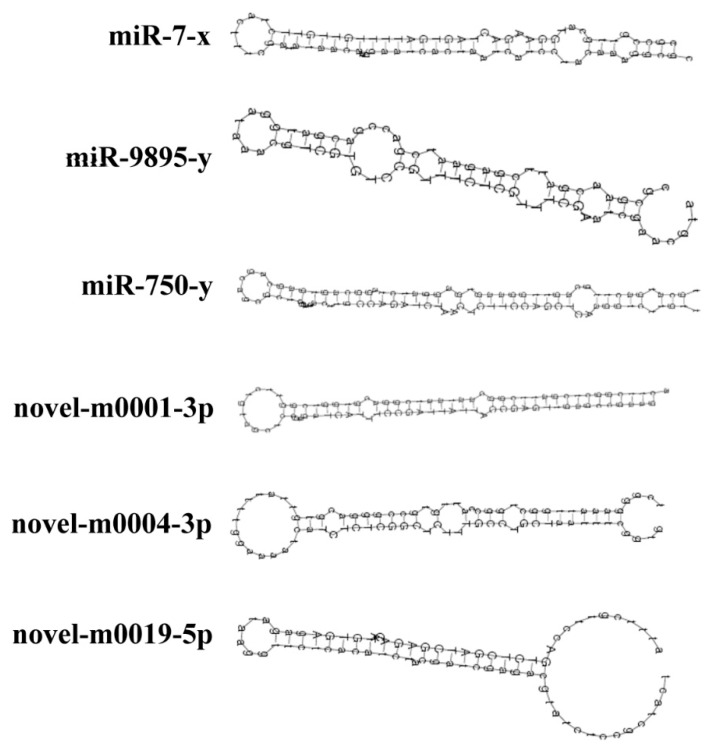
Secondary structures of *A. c. cerana* miRNA precursors. Stem-loop structures of the precursors of miR-7-x, miR-9895-y, miR-750-y, novel-m0001-3p, novel-m0004-3p, and novel-m0009-5p are shown.

To explore known miRNAs and novel miRNAs in the four groups (AmCK1, AmT1, AmCK2, AmT2), the mapped sequences were compared to reference genomes and aligned with known mature miRNAs in the miRBase 21.0 database. In total, 340, 338, 306, and 302 known miRNAs were identified from the AmCK1, AmT1, AmCK2 and AmT2 samples, respectively. These known miRNAs had a broad range of expression levels in *A. c. cerana*, ranging from TPM 90513.14 to TPM 0.38. Among them, bantam-y, miR-184-y, miR-1-y, miR-276-y and miR-750-y were the most abundant known miRNAs in both AcCK1 and AcCK2 (Table S4). We also predicted 25 novel miRNAs not previously found in *A. c. cerana*, including 25, 22, 23 and 17 in AmCK1, AmT1, AmCK2 and AmT2 (Table S5), respectively. Among these, novel-m0019-3p, novel-m0001-3p, novel-m0012-5p, novel-m0004-3p and novel-m0012-3p were the highest-expressed novel miRNAs (Table S5). Moreover, expression clustering analysis showed that these novel miRNAs had various expression levels in different groups (Figure 2). Interestingly, 242 known miRNAs and 23 novel miRNAs were shared by AmCK1 and AmCK2, implying their developmental stage-specific functions. Secondary structures of precursors of three known miRNAs (miR-7-x, miR-9895-y and miR-750-y) and three novel miRNAs (novel-m0001-3p, novel-m0004-3p and novel-m0019-5p) are displayed in Figure 3, and they all have classical Stem-loop structures.

**Figure 4:**
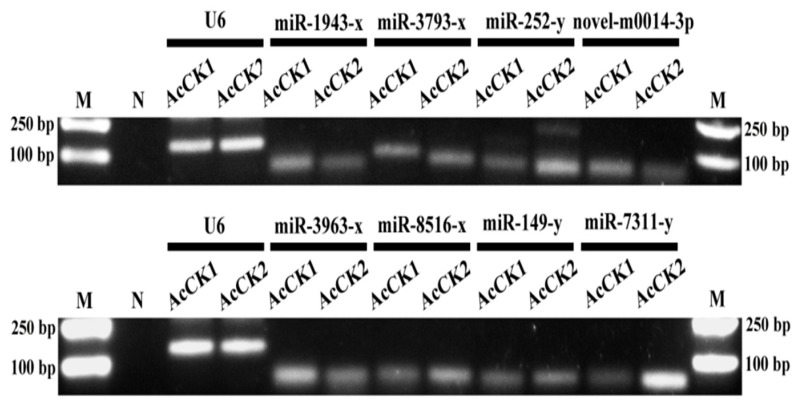
Stem-loop RT-PCR confirmation of eight *A. c. cerana* miRNAs. Lane M: DNA marker; Lane N: Negative control (sterile water was used as PCR template); snRNA U6 was used as positive control. The **upper** panel presents the AGE of PCR amplification products of miR-1943-x, miR-3793-x, miR-252-y, and novel-m0014-3p; the **lower** panel displays the AGE of PCR amplification products of miR-3963-x, miR-8516-x, miR-149-y, and miR-7311-y. The complete gel was presented in Figure S3.

Furthermore, nine miRNAs were selected for Stem-loop RT-PCR validation, the results showed that eight of them yielded signal bands (approximately 60 bp) in both the AcCK1 and AcCK2 samples (Figure 4, see also Figure S3), indicating that most predicted miRNAs were expressed in the *A. c. cerana* worker’s midgut. However, more than one fragment was amplified from miR-1943-x and miR-252-y, suggestive of a non-specific amplification (Figure 4, see also Figure S3).

### 3.3. Differentially Expressed A. c. cerana miRNAs Induced by N. ceranae Invasion

**Figure 5:**
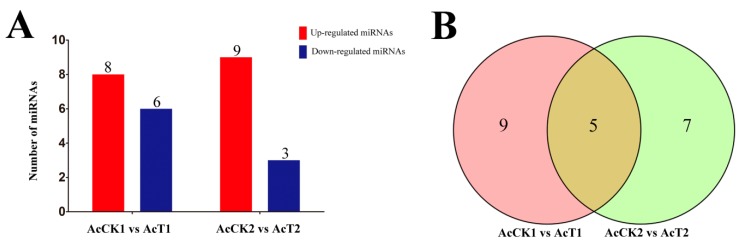
Analysis of DEmiRNAs in *A. c. cerana* workers’ midguts invaded by *N. ceranae*. (**A**) Summary of the number of DEmiRNAs in AcCK1 vs AcT1 and AcCK2 vs AcT2; (**B**) Venn diagram of DEmiRNAs in AcCK1 vs AcT1 and AcCK2 vs AcT2.

A total of 14 DEmiRNAs were detected in AcCK1 vs AcT1 (Table S6), including eight up-regulated and six downregulated miRNAs, while 12 miRNAs with differential expression were identified in AcCK2 vs AcT2 (Table S7), including nine upregulated and three downregulated ones (Figure 5A). Venn diagram analysis was performed, and the result suggested that five DEmiRNAs, including miR-60-y and miR-8462-x, were shared in AcCK1 vs AcT1 and AcCK2 vs AcT2, while nine (e.g., miR-1-x and miR-980-y) and seven (e.g., novel-m0003-3p and miR-92-x) DEmiRNAs were unique for the two comparison groups, respectively (Figure 5B, see also Tables S6 and S7). In addition, more than 85.71% (12) of the DEmiRNAs in AcCK1 vs AcT1 showed a >7-fold difference in gene expression (Table S6), whereas approximately 91.67% (11) of the DEmiRNAs in AcCK2 vs AcT2 displayed a >9-fold differential expression (Table S7). We speculate that these DEmiRNAs are likely to play key roles in the regulation of host responses to *N. ceranae* infection.

### 3.4. Prediction and Enrichment Analysis of the Target mRNAs of A. c. cerana DEmiRNAs

**Figure 6:**
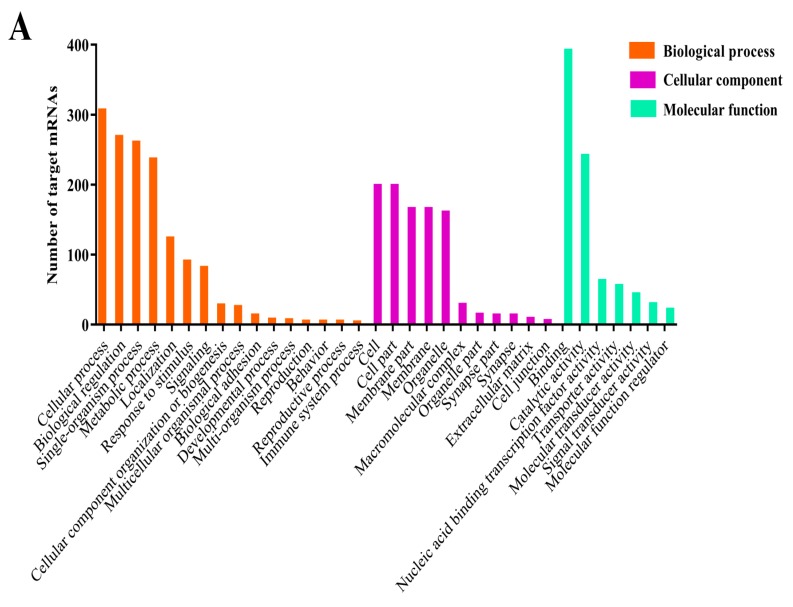

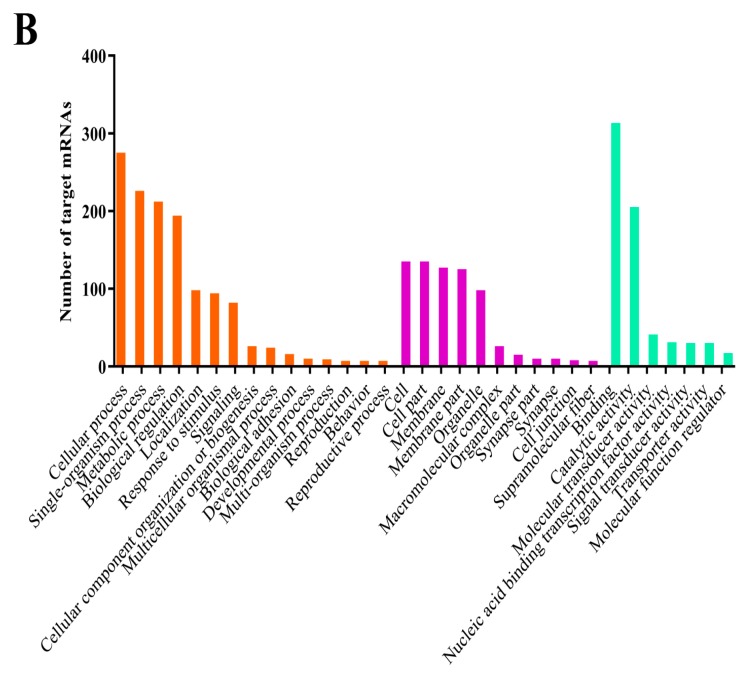
GO categorization of *A. c. cerana* DEmiRNA target mRNAs. (**A**) target mRNAs in AcCK1 vs AcT1; (**B**) target mRNAs in AcCK2 vs AcT2.

DEmiRNAs in AcCK1 vs AcT1 and AcCK2 vs AcT2 were respectively used to search *A. c. cerana* 3’-UTR sequences for predicting potential target mRNAs via a combination of RNAhybrid (v2.1.2) +svm_light (v6.01), Miranda (v3.3a), and TargetScan (v7.0) softwares. In total, 2615 target mRNAs of DEmiRNAs were predicted from AcCK1 vs AcT1, while 1905 DEmiRNA target mRNAs in AcCK2 vs AcT2 were identified. GO classification was conducted, and the results indicated that the target mRNAs of DEmiRNAs in AcCK1 vs AcT1 and AcCK2 vs AcT2 were involved in various biological processes (16 and 15 terms), cellular components (11 and 11 terms), and molecular functions (7 and 7 terms) (Figure 6A,B, see also Tables S8 and S9). Cellular process was the most enriched class in the biological processes; cell and cell part were highly represented in the cellular component categories, and binding and catalytic activity were the top enriched items in molecular functions (Tables S8 and S9). Further investigation indicated that a large quantity of the shared DEmiRNA target mRNAs were engaged in binding, cellular process, single-organism process, metabolic process, biological regulation, and catalytic activity (Tables S8 and S9). However, target mRNAs with the function of extracellular matrix and immune system process were related only in AcCK1 vs AcT1, accounting for three DEmiRNAs including miR-1-x, miR-6313-y, and miR-252-y. Supramolecular fiber function was associated only with AcCK2 vs AcT2, accounting for novel-m0003-3p.

**Figure 7:**
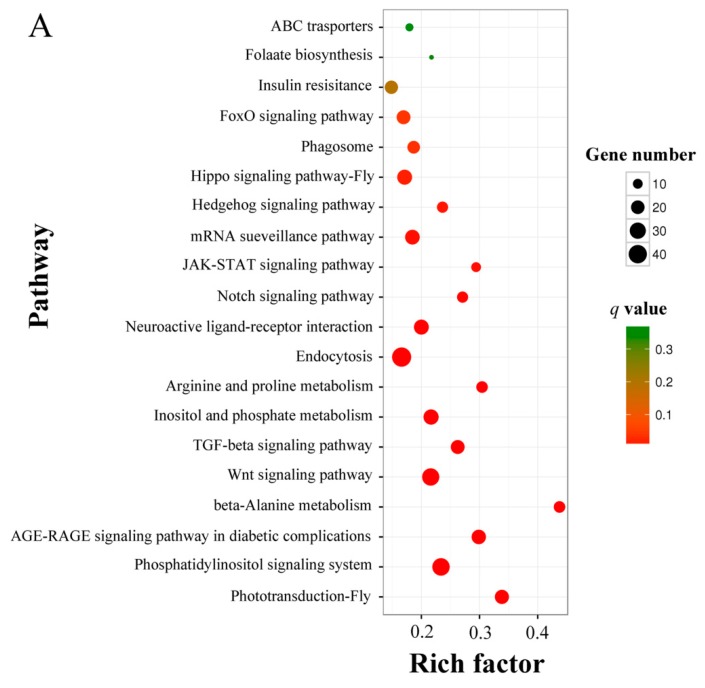

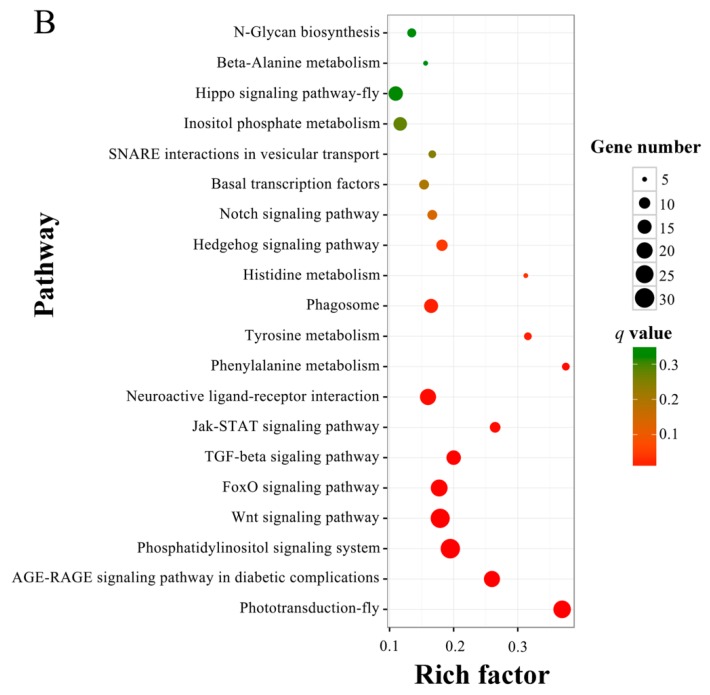
KEGG pathway enrichment analysis for *A. c. cerana* DEmiRNA target mRNAs. (**A**) target mRNAs in AcCK1 vs AcT1; (**B**) target mRNAs in AcCK2 vs AcT2.

KEGG enrichment analysis for DEmiRNA-targeted genes was conducted, and the results demonstrated that the target mRNAs of shared DEmiRNAs in AcCK1 vs AcT1 were connected with 104 pathways; amongst them, the highest enriched pathways were endocytosis, the phosphatidylinositol signaling system, the Wnt signaling pathway, inositol phosphate metabolism, and neuroactive ligand-receptor interaction (Figure 7A, see also Table S10). The target mRNAs of DEmiRNAs in AcCK2 vs AcT2 were involved in 92 pathways, and the most enriched pathway was the phosphatidylinositol signaling system, followed by the Wnt signaling pathway, phototransduction, endocytosis and the FoxO signaling pathway (Figure 7B, see also Table S11). Interestingly, 62 and 52 metabolism-related pathways were respectively enriched by 234 and 195 target mRNAs in AcCK1 vs AcT1 and AcCK2 vs AcT2, and, in addition, there were five and six target mRNAs enriched in development in these two comparison groups (Table S10 and Table S11). These results demonstrated that DEmiRNAs could regulate the growth, development and metabolism of *A. c. cerana* workers’ midguts.

### 3.5. Regulatory Networks between A. c. cerana DEmiRNAs and Their Target mRNAs

**Figure 8:**
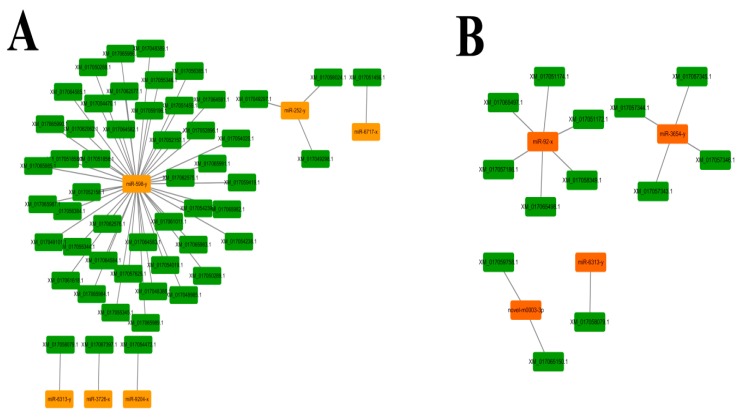
Regulation networks between *A. c. cerana* DEmiRNA and their target mRNAs. (**A**) DEmiRNA-target mRNA regulation network in AcCK1 vs AcT1; (**B**) DEmiRNA-target mRNA regulation network in AcCK2 vs AcT2.

Cytoscape was employed for visualization of the regulatory networks between DEmiRNA and their target mRNAs. A single mRNA can be targeted by various miRNAs, whereas a single miRNA is also capable of targeting different mRNAs [57]. In these regulatory networks, it can be clearly seen that DEmiRNAs and their target mRNAs in AcCK1 vs AcT1 formed complex networks. MiR-598-y had as many as 45 target mRNAs, and miR-252-y bound to three targets including XM_017049297.1, XM_017049298.1 and XM_017058024.1 (Figure 8A). In contrast, miR-6313-y, miR-3726-x, miR-9204-x and miR-6717-x could bind to only one target (Figure 8A). In addition, as shown in Figure 8B, for DEmiRNAs in AcCK2 vs AcT2, miR-92-x, miR-3654-y, novel-m0003-3p and miR-6313-y linked to six, four, two and one target mRNAs, respectively.

### 3.6. Verification of A. c. cerana DEmiRNAs via RT-qPCR

**Figure 9:**
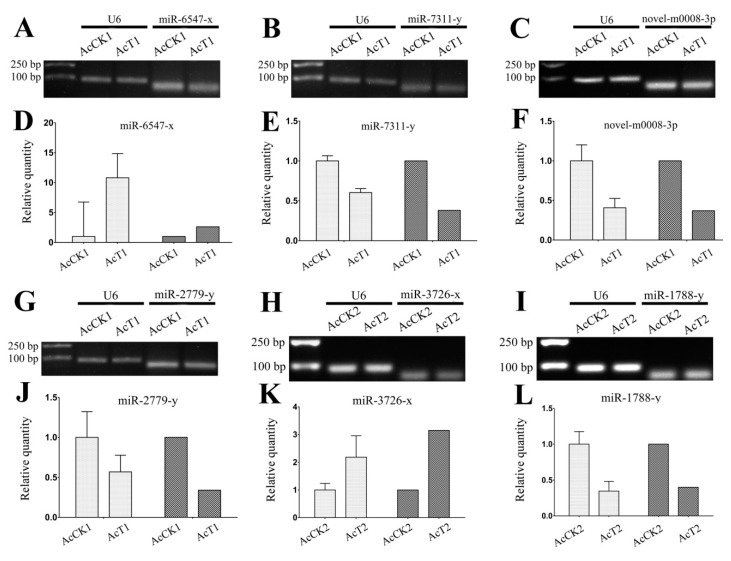

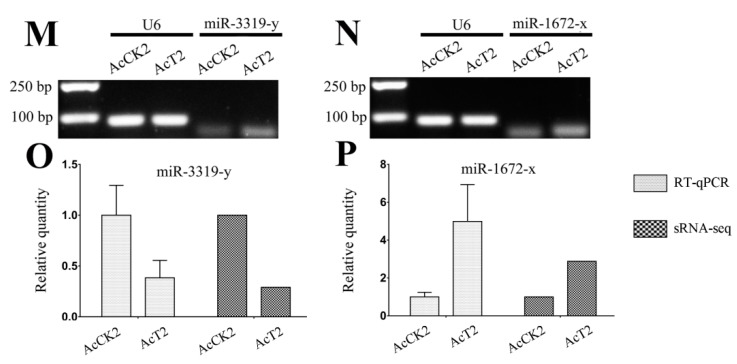
Stem-loop RT-PCR and RT-qPCR validation of *A. c. cerana* DEmiRNAs. (**A**–**C**) Stem-loop RT-PCR validation of miR-6547-x, miR-7311-x, and novel-m0008-3p; (**G**–**I**) Stem-loop RT-PCR validation of miR-2779-y, miR-3726-x, and miR-1788-y; (**M**,**N**) Stem-loop RT-PCR validation of miR-3319-y and miR-1672-x; (**D**–**F**) RT-qPCR validation of miR-6547-x, miR-7311-x, and novel-m0008-3p; (**J**–**L**) RT-qPCR validation of miR-2779-y, miR-3726-x, and miR-1788-y; (**O**,**P**) RT-qPCR validation of miR-3319-y and miR-1672-x; snRNA U6 was used as a positive control in Stem-loop RT-PCR and reference gene in RT-qPCR. The experiment was performed three times using three independent biological samples. The left two charts showed the result of RT-qPCR of selected DEmiRNA (in light gray), while the right two charts showed the differential expression (log_2_(Fold change)) of the same selected DEmiRNA from sRNA-seq data (in dark gray). RT-qPCR data are represented as the mean ± SD.

To verify the high-throughput sequencing data in our study, the expression levels of nine randomly selected DEmiRNAs in AcCK1 vs AcT1 and AcCK2 vs AcT2 were examined by RT-qPCR. The expression trends of eight DEmiRNAs in the two comparison groups were roughly the same as the result of the sRNA-seq (Figure 9), indicative of the accuracy and reliability of our sequencing data.

## 4. Discussion

Previous studies have mainly been focused on western honeybee miRNAs [58,59,60,61,62]. However, relevant studies on the eastern honeybee were extremely limited. Shi et al. previously performed Illumina sequencing of miRNAs in the royal jelly from *A. mellifera* and *A. cerana*, and the results showed that there were differences between them; they further found that the transcriptomes of *A. mellifera* adults were affected by the two kinds of royal jelly [63]. Over the years, increasing evidence has demonstrated that miRNAs play vital roles in host-pathogen interactions via regulation of the gene expression of hosts or pathogens [64,65,66]. Although advances have been made regarding the actions of miRNAs in the interactions between the western honeybee and *N. ceranae* [67,68], the miRNA profiles and DEmiRNAs in eastern honeybee-*N. ceranae* interactions remains completely unknown. In a previous study, Huang et al. deep-sequenced *A. mellifera* miRNAs daily across the six-day reproductive cycle of *N. ceranae* and identified 17 DEmiRNAs that target more than 400 genes [28]. Here, in order to identify miRNAs involved in the eastern honeybee responding to *N. ceranae* infection, we constructed for the first time 12 small RNA libraries from untreated and *N. ceranae*-treated *A. c. cerana* workers’ midgut samples. In total, 529 known and 25 novel miRNAs were identified from sRNA-seq data using bioinformatics, which enriches the miRNA reservoir of the eastern honeybee and provides candidate miRNAs for functional studies in the future. In addition, eight predicted miRNAs were confirmed to be expressed in the midguts of *A. c. cerana* workers via Stem-loop RT-PCR (Figure 4). Additionally, we also validated the expression of another eight DEmiRNAs (Figure 9A–C,G–I,M,N). Deep sequencing has become the mainstream method to explore novel miRNAs at present [69]. Notably, all of the 25 novel miRNAs predicted in this work were weakly expressed, in accordance with the results for some lower invertebrates such as *Drosophila* and *Cyprinus carpio* [57,70]. In our study, the most abundant miRNAs in AcCK1 and AcCK2 were bantam-y, miR-184-y, miR-1-y, miR-276-y, miR-750-y, miR-3477-x, miR-283-x, miR-12-z, miR-31-x and miR-9-z with TPM ≥ 31,625.97 each (Table S4). Surprisingly, these miRNAs were also highly expressed in AcT1 and AcT2 (Table S4). As a versatile player in many biological processes in *Drosophila*, *bantam* regulates fly development [71,72], cell proliferation and apoptosis in many cell types [73,74], and the production of the molting hormone ecdysone [75]. Through systematic manipulation of Dpp signaling in the *Drosophila* wing, Zhang et al. discovered that Dpp promoted proliferation in the lateral wing disc and repressed proliferation in the medial wing disc via *omb*, which controlled the regional proliferation rate by oppositely regulating transcription of *bantam* in the medial versus lateral wing disc [71]. Wu et al. previously investigated the role of the *bantam* miRNA in the regulation of neuroblast homeostasis in the *Drosophila* brain and found that *bantam* is a direct transcriptional target of the Notch signaling pathway; in addition, bantam feedback regulates the pathway by negatively regulating its target mRNA *numb* [72]. In the current work, *bantam*-y was the miRNA with the highest expression level in both AcCK1 and AcCK2 (Table S4), implying its key role in the regulation of the development of the *A. c. cerana* workers’ midguts. MiR-2, an invertebrate-specific miRNA family that has been predicted in fruit flies, is highly expressed in the heads of *Drosophila* and *Bombyx mori* [76,77] and the neurons in *Caenorhabditis* [78]. It has also been shown to participate in the regulation of apoptosis of neuroblasts during the normal development of the nervous system in *Drosophila* [79]. Recent evidence shows that miR-2 regulates functions essential for the normal wing morphogenesis of *B. mori* via targeting of *awd* and *fng* [80]. In the embryonic development of *Drosophila*, miRs-2/13, including miR-2a, miR-2b, miR-13a, and miR-13b, play an important regulatory part in the embryonic development of *Drosophila* by targeting nine genes, including *Sos* and *Myd88* [81]. In the present study, miR-2-y and miR-13-y were detected to be highly expressed in the normal midguts of *A. c. cerana* workers (Table S4), which suggests these two miRNAs may play a fundamental role in regulating the development and morphogenesis of the midgut and apoptosis of the midgut cells. However, the functions of other miRNAs that were abundant in *A. c. cerana* midgut samples, such as miR-750-y and miR-3477-x, remain largely unknown.

DEmiRNAs in the midguts of *A. c. cerana* workers challenged by *N. ceranae* are particularly worthy of attention since they are likely to be direct regulators of host-pathogen interactions. In this research, 14 and 12 DEmiRNAs were identified in AcCK1 vs AcT1 and AcCK2 vs AcT2, respectively. Among them, miR-60-y, miR-8462-x, miR-2965-y, miR-676-y, and miR-6313-y were shared by the two comparison groups, implying their primary roles in host responses to *N. ceranae* infections. In addition, nine DEmiRNAs, including miR-1-x, miR-980-y, miR-965-x, miR-598-y, miR-6717-x, miR-4635-y, miR-9204-x, miR-3726-x, and miR-252-y, were specific in AcCK1 vs AcT1, and all of them were known miRNAs (Figure 5B, see also Tables S6 and S7). Additionally, seven DEmiRNAs, including two novel miRNAs (novel-m0003-3p and novel-m0019-5p), were specific in AcCK2 vs AcT2 (Figure 5B, see also Tables S6 and S7). We believe these unique DEmiRNAs play specific parts during different stages of host responses under the stress of *N. ceranae*.

To gain further insight into the potential genes associated with *N. ceranae* infection, we conducted GO analysis for DEmiRNA target mRNAs to identify biologically important terms. The mostly enriched terms for the targets in AcCK1 vs AcT1 and AcCK2 vs AcT2 were binding, cellular process, biological regulation, single-organism process, and catalytic activity (Figure 6, see also Tables S8 and S9), indicating the regulation of host metabolism and cellular activity via DEmiRNAs in response to *N. ceranae*. Huang and colleagues found that the target mRNAs of DEmiRNAs in the *N. ceranae*-infected midgut of the *A. mellifera* workers were mostly involved in binding, signaling, nucleus, transmembrane transport, and DNA binding, which differ from our findings in the present study [28]. This difference reflects the different responses of the two honeybee species with different resistances to the same fungal pathogen, *N. ceranae*. Considering the fact that *A. cerana* is more resistant to *N. ceranae* compared to *A. mellifera*, we inferred that DEmiRNAs may in part play a special role in the *N. ceranae*-resistance difference. In addition, we found 88 and 89 DEmiRNA-targeted mRNAs in AcCK1 vs AcT1 and AcCK2 vs AcT2 were respectively enriched in response to stimulus, and there was one target mRNA for DEmiRNAs in AcCK1 vs AcT1 enriched in immune system process, which indicates the regulatory roles of the corresponding DEmiRNAs in host immunity defenses against *N. ceranae*. Furthermore, KEGG analysis for the target mRNAs was performed to investigate the pathways influenced by *N. ceranae*. The result showed that out of the 104 pathways enriched by target mRNAs of DEmiRNAs in AcCK1 vs AcT1, 62 were related to material and energy metabolism, such as carbohydrate metabolism including the pentose phosphate pathway and citrate cycle, lipid metabolism including arachidonic acid metabolism and fatty acid biosynthesis, nucleotide metabolism including purine metabolism and pyrimidine metabolism, energy metabolism including sulfur metabolism and oxidative phosphorylation (Table S10). A total of 92 pathways were enriched by DEmiRNA target mRNAs in AcCK2 vs AcT2; among them, 52 pathways were associated with material and energy metabolism (Table S11). These results demonstrated that host DEmiRNAs regulate metabolism-related genes in response to *N. ceranae* invasion. Meanwhile, immunity-related pathways were further analyzed, and the results showed that endocytosis, phagosome, lysosome, ubiquitin mediated proteolysis, Jak-STAT signaling pathway, and MAPK signaling pathways were enriched by the target mRNAs in AcCK1 vs AcT1 (Table S10). Surprisingly, these immune pathways were also enriched by DEmiRNA target mRNAs in AcCK2 vs AcT2 (Table S11). These results demonstrated that DEmiRNAs and their target mRNAs were involved in cellular and humoral immune responses of the host to *N. ceranae* invasion. Together, these findings indicate that host material and energy metabolism, cellular activity, and immunity defenses were significantly impacted by *N. ceranae* infection, and DEmiRNAs played a comprehensive role in host-pathogen interactions.

MiR-1 is a muscle-specific miRNA that plays important roles in regulating heart development and muscle differentiation [82,83]. It has also been known to be a tumor suppressor gene that not only inhibits cancer cell proliferation, metastasis, and invasion, but also enhances apoptosis by regulating oncogenic targets in many cancer cells [84,85]. In insects, miR-1 was found to be involved in immune processes in *Drosophila melanogaster* [86] and *Aedes aegypti* [87]. Besides, Huang et al. suggested a significant down-regulation of ame-miR-1 in *Apis mellifera* workers at 6 dpi with *N. ceranae* [25]. In this work, miR-1-x was dramatically down-regulated in AcCK1 vs AcT1, suggesting that the host and fungal pathogen can interact with each other via miR-1-x by regulating host proliferation and apoptosis and immune response. Considering the conservation between miR-1-x and ame-miR-1, we speculated that miR-1 family participates in honeybee response to *N. ceranae* invasion as a general regulator. Wu et al. carried out Illumina sequencing of dengue virus-2 (DENV-2)-infected and uninfected *Aedes albopictus* and observed a series of DEmiRNAs including miR-252 [88]. By using RT-qPCR, Yan et al. detected a high expression level of miR-252 in DENV-2-infected C6/36 cells and mosquitoes compared with uninfected cells and mosquitos and further revealed that DENV-2 envelope protein expression can be affected by the overexpression of miR-252 with a mimic or down-regulation using an inhibitor [89]. Recently, Lim and colleagues discovered that miR-252-5p can control the cell cycle by directly repressing Abelson interacting protein (Abi) in *Drosophila* S2 cells by using cross-linking immunoprecipitation and deep sequencing of endogenous Argonaute 1 (Ago1) protein [90]. In our study, miR-252-y had a weak up-regulation in the midgut 7 dpi with *N. ceranae*, suggesting that miR-252-y may participate in *N. ceranae* defense in *A. c. cerana*. As a member of the miR-17-92 cluster, miR-92a inhibits apoptosis and promotes the proliferation of other cell types, including endothelial cells [91,92]. Kurze et al. first showed the importance of apoptosis for *N. ceranae* infections [93]. In addition, apoptosis is of great importance for pathogen defense in multicellular organisms including *B. mori* [94]. Previous studies have demonstrated that *Spodoptera frugiperda* larvae and *B. mori* larvae battle baculovirus infection by selective apoptosis of infected cells from the midgut epithelium [95,96]. *N. ceranae* is able to enhance its development during the infection process by preventing apoptosis in epithelial cells of infected *A. mellifera* [97]. A previous study showed that miR-194 expression was markedly decreased in A549 alveolar epithelial cells following infection with influenza A virus (IAV), and miR-194 could suppress fibroblast growth factor 2 (FGF2) expression at the mRNA and protein levels [98]. Xie and colleagues suggested that miR-194 increases cell apoptosis by inhibiting the NF-*κ*B pathway in WI38 cells, and it may be used as a potential targeted therapy for the treatment of infantile pneumonia [99]. In this present study, miR-92-x had a sharp down-regulation, while miR-194-y was significantly up-regulated in the *A. c. cerana* worker’s midgut at 10 dpi with *N. ceranae*. Therefore, we inferred that *A. c. cerana* could fight *N. ceranae* by promoting apoptosis of host midgut cells via regulation of the expression levels of miR-92-x and miR-194-y. Further studies are needed to confirm the mechanism underlying the roles of above-mentioned DEmiRNAs in host-pathogen interactions. Recently, Evans and Huang discovered both *N. ceranae* and *A. mellifera* gene expression were altered by knockdown of *Dicer* gene of *N. ceranae*, and further found parasite miRNAs may regulate host metabolism and immune response via cross-talk with *A. mellifera* mRNAs [68]. It’s known that complex interactions exist between honeybee and microsporidian [28,67,68]. Thus, whether *A. c. cerana* miRNAs identified in this study can regulate *N. ceranae* mRNAs, and whether *N. ceranae* miRNAs could regulate *A. c. cerana* mRNAs, is a very interesting research direction.

A single miRNA can regulate several target mRNAs at the same time to inhibit their expression and vice versa [100]. MiR-598 has been found to play inhibitory role in osteosarcoma progression in vivo and in vitro by modulating osteoblastic differentiation in the microenvironment and targeting *PDGFB* and *MET*. Based on expressed sequence tags (ESTs) resources from the LNCaP cells, Saravanan et al. identified an hsa-miR-3654 with a higher expression level in LNCaP cells than the normal and androgen insensitive prostate cancer cell lines (PNT1A, PC-3) [101]. To our knowledge, there is no documentation of miR-598 and miR-3654 in insects until now. In the current work, miR-598 lies in the center of the regulation networks of DEmiRNAs in AcCK1 vs AcT1, linking to as many as 45 target mRNAs such as XM_017048388.1, XM_017049101.1, and XM_017051459.1 (Figure 8A), while miR-3654-y was a key miRNA in the regulation networks of DEmiRNAs in AcCK2 vs AcT2, binding to four targets including XM_017057343.1, XM_017057344.1, XM_017057345.1, and XM_017057346.1 (Figure 8B). These results indicated that miR-598 and miR-3654-y are likely to be key regulators of the interactions between *A. c. cerana* and *N. ceranae*, and these two miRNAs would be our candidates for future studies of host immune response and resistance to *N. ceranae*.

In conclusion, using high-throughput sequencing technology and bioinformatics, we systematically investigated the highly expressed miRNAs in the normal *A. c. cerana* midgut, and DEmiRNAs as well as their target mRNAs in the *N. ceranae*-infected midgut. This study provides the first global view of the miRNA profiles and DEmiRNAs in the midgut of the *A. c. cerana* worker invaded by *N. ceranae*. Findings in the present study demonstrated that *N. ceranae* invasion causes alterations in the expression of miRNAs that regulate metabolism, cellular activity, and immune response in the host. Taken together, our results not only provide novel insights into understanding *A. c. cerana*-*N. ceranae* interactions but also lay a foundation for deciphering the molecular mechanisms underlying the resistance of the eastern honeybee to *N. ceranae*.

## 5. Conclusions

In a nutshell, this is the first study on eastern honeybee miRNA response to *N. ceranae* infection from a genome-wide perspective, which demonstrates the expression pattern of miRNAs in *A. c. cerana* workers was altered by *N. ceranae* invasion. We identified 579 known miRNAs and 25 novel miRNAs in the midguts of *A. c. cerana* workers, and showed that 14 and 12 DEmiRNAs were *N. ceranae*-responsive in midguts at 7 dpi and 10 dpi, respectively. DEmiRNAs may play an important role in the host stress responses including immune responses via negatively regulate target mRNAs. Our results provide a rich genetic resource and potential candidates for further investigation of the regulatory roles of miRNAs in host responses to *N. ceranae*. Furthermore, the study may help elucidating the complex molecular mechanisms underlying the eastern honeybee-microsporidian interactions.

## Figures and Tables

**Table 1:** Summary of small RNA sequencing datasets from untreated and *N. ceranae*-treated midgut samples of *A. c. cerana*.

Sample	Raw Reads	Clean Reads	Clean Tags
AcCK1-1	12,757,706	12,049,987 (94.45%)	10,213,625 (84.76%)
AcCK1-2	12,794,543	12,122,624 (94.75%)	10,275,851 (84.77%)
AcCK1-3	11,328,883	10,727,931 (94.70%)	8,671,328 (80.83%)
AcCK2-1	17,161,911	16,292,122 (94.93%)	14,528,074 (89.17%)
AcCK2-2	11,666,305	11,306,117 (96.91%)	9,862,922 (87.24%)
AcCK2-3	11,757,223	11,328,016 (96.35%)	9,310,382 (82.19%)
AcT1-1	11,213,906	10,923,950 (97.41%)	9,356,024 (85.65%)
AcT1-2	14,549,245	13,778,004 (94.70%)	11,661,491 (84.64%)
AcT1-3	11,029,767	10,800,311 (97.92%)	8,914,299 (82.54%)
AcT2-1	13,263,930	12,775,381 (96.32%)	10,974,972 (85.91%)
AcT2-2	13,688,082	13,150,156 (96.07%)	10,977,135 (83.48%)
AcT2-3	12,936,357	12,467,737 (96.38%)	10,859,224 (87.10%)

## Supplementary Materials

The following are available online at https://www.mdpi.com/2075-4450/10/9/258/s1, Figure S1. Validation of purified *N. ceranae* spores and inoculation of *A. c. cerana* worker. (**A**) Microscopic observation of purified *N. ceranae* spores (400× amplification); (**B**) Artificial inoculation of *A. c. cerana* worker with *N. ceranae* spores; (**C**) RT-PCR detection of sucrose solution containing *N. ceranae* spores; Lane M: DNA marker; Lane NC: *N. ceranae* specific primers (positive control), a signal fragment (about 76 bp) was amplified; Lane N: Sterile water (negative control); Lane NA: *N. apis* specific primers (negative control); Lane ABPV: ABPV specific primers; Lane BQCV: BQCV specific primers; Lane DWV: DWV specific primers; Lane SBV: SBV specific primers; Lane CBPV: CBPV specific primers; Lane KBV: KBV specific primers; Lane IAPV: IAPV specific primers, Figure S2. Pearson correlations between different biological replicas within each sample group of *A. c. cerana* workers’ midguts, Figure S3. Aarose gel electrophoresis of Stem-loop RT-PCR products from eight *A. c. cerana* miRNAs, Table S1. Primers for RT-PCR detection of sucrose solution containing *N. ceranae* spores, Table S2. Primers for Stem-loop RT-PCR and RT-qPCR in this study, Table S3. Small RNA profiling and distribution among four *A. c. cerana* worker’s midgut samples, Table S4. Top 20 highest-expressed known miRNAs in *A. c. cerana* worker’s midgut, Table S5. The expressed novel miRNAs in *A. c. cerana* worker’s midgut, Table S6. Summary of DEmiRNAs in AcCK1 vs AcT1 comparison group, Table S7. Summary of DEmiRNAs in AcCK2 vs AcT2 comparison group, Table S8. GO enrichment analysis for the target mRNAs of DEmiRNAs in AcCK1 vs AcT1, Table S9. GO enrichment analysis for the target mRNAs of DEmiRNAs in AcCK2 vs AcT2, Table S10. KEGG pathways enriched by the target mRNAs of DEmiRNAs in AcCK1 vs AcT1, Table S11. KEGG pathways enriched by the target mRNAs of DEmiRNAs in AcCK2 vs AcT2.
