# Supplementary figures and images for: Comparative Identification of MicroRNAs in Apis cerana cerana Workers’ Midguts in Response to Nosema ceranae Invasion
Source: Insects. 2019 Aug 21;10(9):258. doi: 10.3390/insects10090258 (PMC6780218; doi:10.3390/insects10090258)

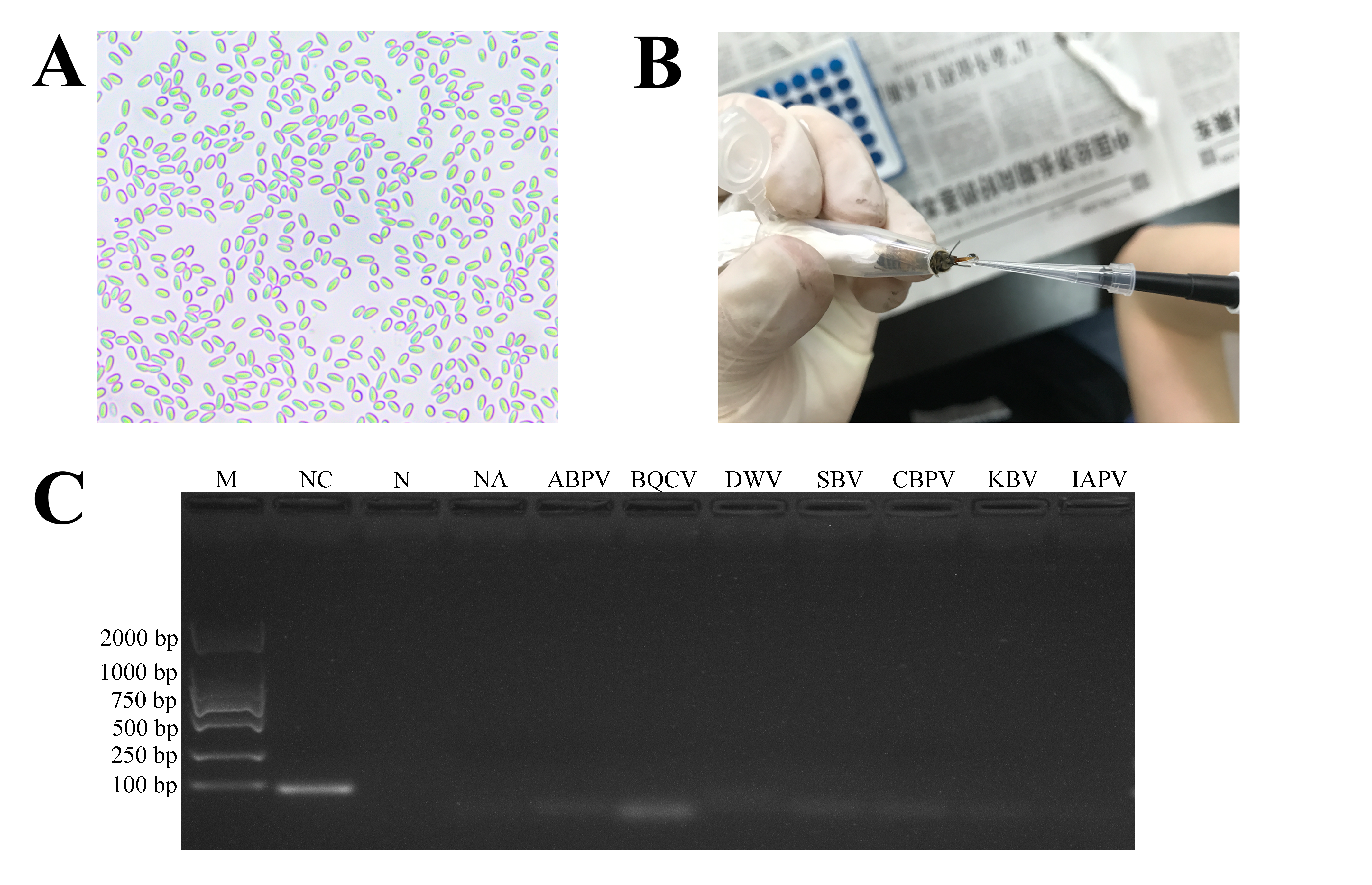

Supplement: Supplementary file 1 [file insects-10-00258-s001.zip › Supplementary Files/Figure S1.tif]

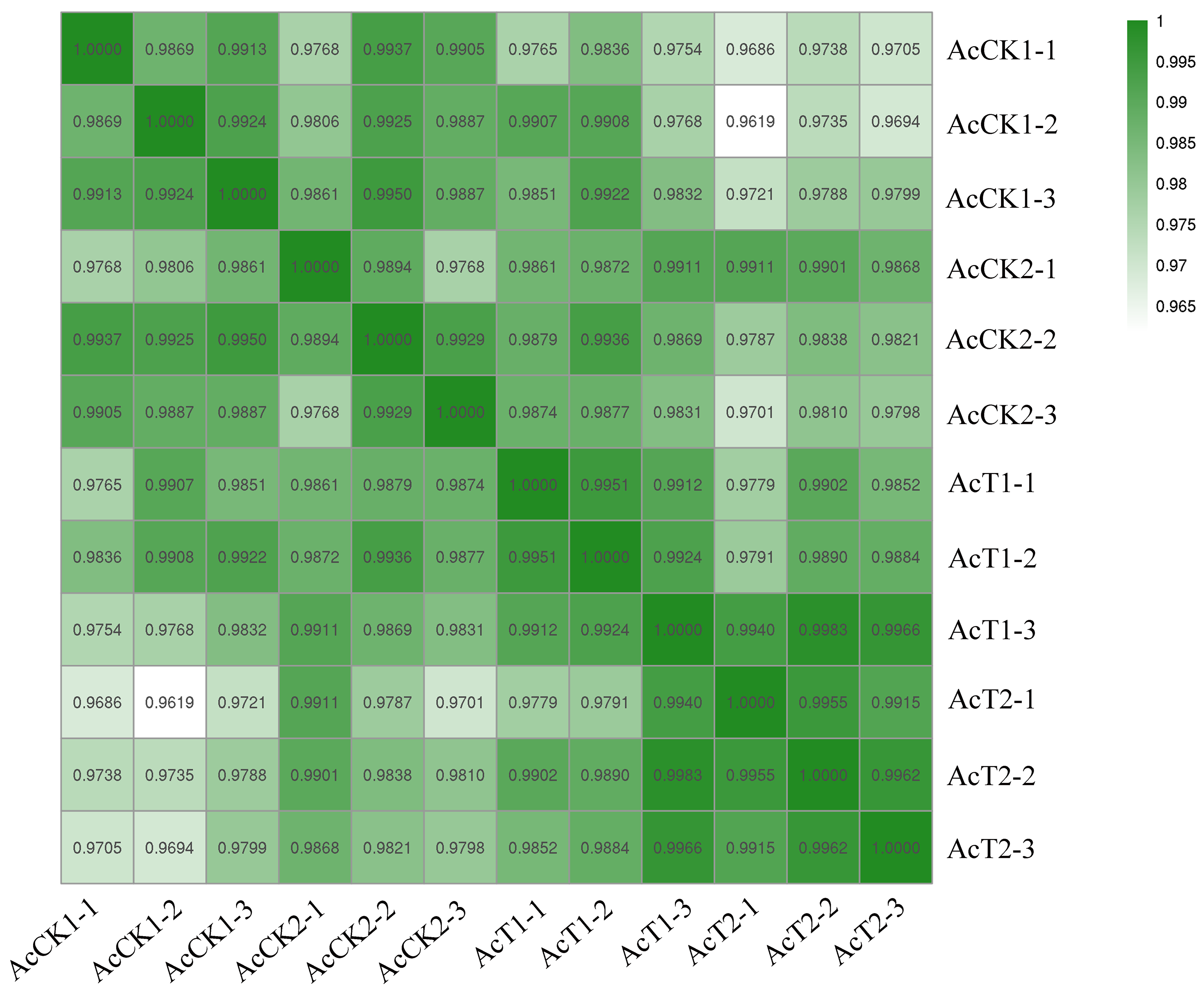

Supplement: Supplementary file 1 [file insects-10-00258-s001.zip › Supplementary Files/Figure S2.tif]

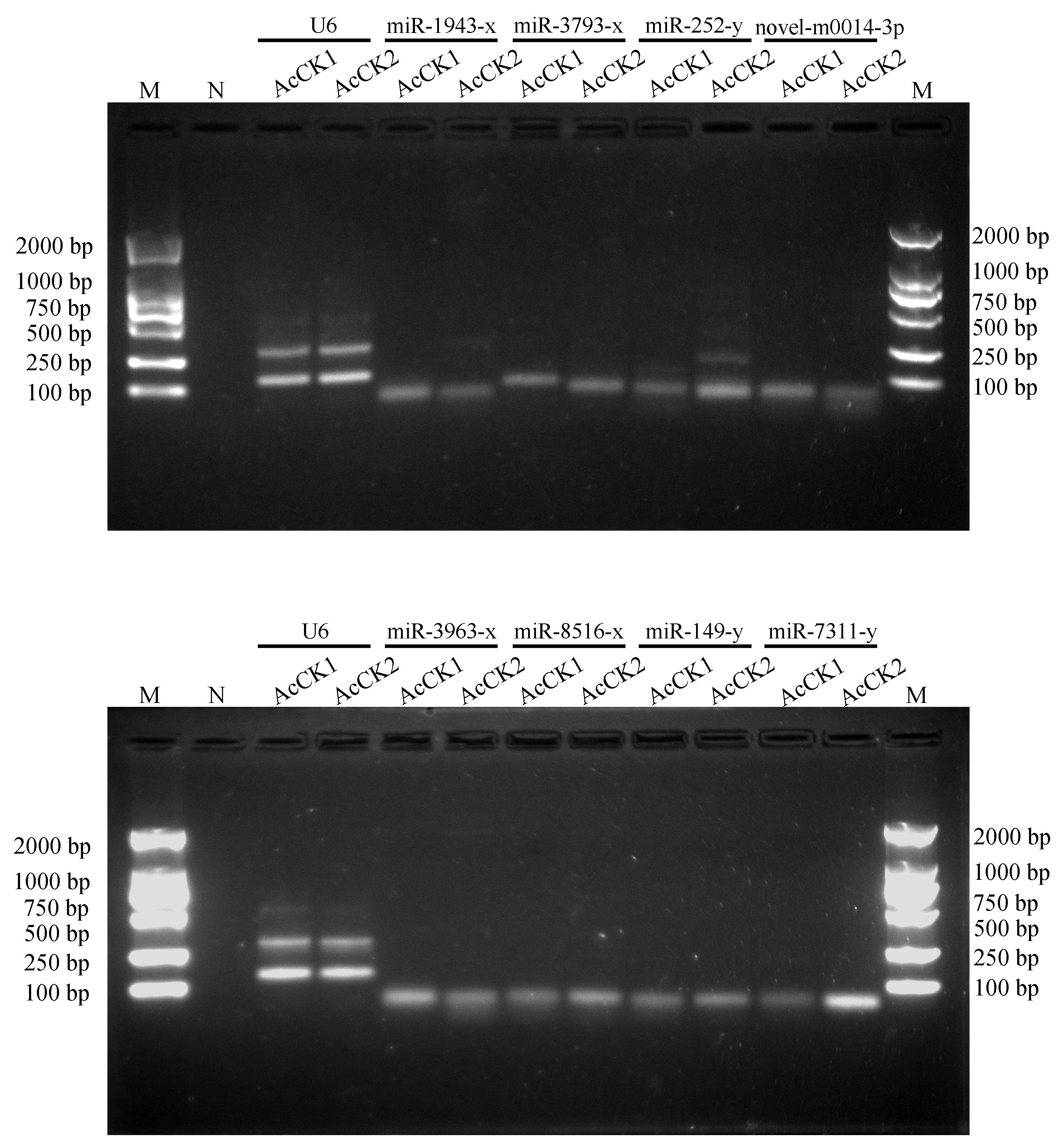

Supplement: Supplementary file 1 [file insects-10-00258-s001.zip › Supplementary Files/Figure S3.tif]
