# Supplementary material for: Comparative Identification of MicroRNAs in Apis cerana cerana Workers’ Midguts in Response to Nosema ceranae Invasion
Source: Insects. 2019 Aug 21;10(9):258. doi: 10.3390/insects10090258 (PMC6780218; doi:10.3390/insects10090258)
Supplement: Supplementary file 1 [file insects-10-00258-s001.zip › Supplementary Files/Table S1.doc]

**Table S1.**  Primers for RT-PCR detection of sucrose solution containing *N. ceranae* spores*.*

| **Primer name** | **Sequence (5’-3’)** | **Product size (bp)** | **Reference** |
| --- | --- | --- | --- |
| NC-F | CGAGCGGTTTCCCATCTCAGTA | 76 | This study |
| NC-R | AAAACACCGGAACTCGTCAGCT |  |  |
| NA-F | CCATTGCCGGATAAGAGAGT | 401 | [15] |
| NA-R | CACGCATTGCTGCATCATTGAC |  |  |
| ABPV-F | TTATGTGTCCAGAGACTGTATCCA | 900 | [33] |
| ABPV-R | GCTCCTATTGCTCGGTTTTTCGGT |  |  |
| BQCV-F | TGGTCAGCTCCCACTACCTTAAAC | 700 | [33] |
| BQCV-R | GCAACAAGAAGAAACGTAAACCAC |  |  |
| DWV-F | TCCATCAGGTTCTCCAATAACGG | 451 | [34] |
| DWV-R | CCACCCAAATGCTAACTCTAACGC |  |  |
| CBPV-F | AGTTGTCATGGTTAACAGGATACGAG | 455 | [35] |
| CBPV-R | TCTAATCTTAGCACGAAAGCCGAG |  |  |
| KBV-F | GATGAACGTCGACCTATTGA | 417 | [36] |
| KBV-R | TGTGGGTTGGCTATGAGTCA |  |  |
| IAPV-F | GGTCCAAACCTCGAAATCAA | 840 | [37] |
| IAPV-R | TTGGTCCGGATGTTAATGGT |  |  |
| SBV-F | GCACGTTTAATTGGGGATCA | 693 | [37] |
| SBV-R | CAGGTTGTCCCTTACCTCCA |  |  |

NC: *Nosema ceranae*; NA: *Nosema apis*; ABPV: acute bee paralysis virus; BQCV: black queen cell virus; DWV: deformed wing virus; CBPV: chronic bee paralysis virus; KBV: Kashmir Bee Virus; IAPV: israeli acute paralysis virus; SBV: sacbrood virus
