# Supplementary material for: Comparative Identification of MicroRNAs in Apis cerana cerana Workers’ Midguts in Response to Nosema ceranae Invasion
Source: Insects. 2019 Aug 21;10(9):258. doi: 10.3390/insects10090258 (PMC6780218; doi:10.3390/insects10090258)
Supplement: Supplementary file 1 [file insects-10-00258-s001.zip › Supplementary Files/Table S3.doc]

Table S3. Small RNA profiling and distribution among four *A. c. cerana* worker's midgut samples.

| **Types** | **AmCK1** | **AmT1** | **AmCK2** | **AmT2** |
| --- | --- | --- | --- | --- |
| Total RNA | 9720268 | 9977271 | 11233793 | 10937110 |
| miRNA | 978435 | 577121 | 608488 | 286809 |
| tRNA | 192134 | 101602 | 221407 | 146370 |
| rRNA | 5054110 | 3611375 | 4923233 | 4631227 |
| snRNA | 11137 | 5901 | 6620 | 5123 |
| snoRNA | 244 | 332 | 176 | 584 |
| Exon:+ | 15918 | 6798 | 9246 | 11943 |
| Exon:- | 88024 | 51035 | 40587 | 26733 |
| Intron:+ | 19686 | 10076 | 14761 | 12624 |
| Intron:- | 2979 | 2330 | 2681 | 3447 |
| Repeat | 457936 | 293249 | 415936 | 415268 |
| Unanotated | 3300268 | 5574461 | 5370922 | 5793014 |
