# Supplementary material for: Comparative Identification of MicroRNAs in Apis cerana cerana Workers’ Midguts in Response to Nosema ceranae Invasion
Source: Insects. 2019 Aug 21;10(9):258. doi: 10.3390/insects10090258 (PMC6780218; doi:10.3390/insects10090258)
Supplement: Supplementary file 1 [file insects-10-00258-s001.zip › Supplementary Files/Table S4.doc]

Table S4. Top 20 highest-expressed known miRNAs in *A. c. cerana* worker's midgut.

| **miRNA** | **Length** | **Sequence** | **AcCK1**  **TPM** | **AcT1**  **TPM** | **AcCK2**  **TPM** | **AcT2**  **TPM** |
| --- | --- | --- | --- | --- | --- | --- |
| bantam-y | 23 | TGAGATCATTGTGAAAGCTGATT | 90513.74 | 79896.49 | 82999.18 | 77820.64 |
| miR-184-y | 21 | TGGACGGAGAACTGATAAGGG | 67672.30 | 72976.81 | 67645.90 | 78798.41 |
| miR-1-y | 22 | TGGAATGTAAAGAAGTATGGAG | 57597.89 | 70322.89 | 63024.94 | 75316.67 |
| miR-276-y | 22 | TAGGAACTTCATACCGTGCTCT | 67552.20 | 58963.28 | 57639.96 | 48382.65 |
| miR-750-y | 23 | CCAGATCTAACTCTTCCAGCTCA | 64499.40 | 56348.96 | 60514.40 | 48895.42 |
| miR-3477-x | 23 | TAATCTCATGCGGTAACTGTGAG | 53614.50 | 54192.60 | 47257.80 | 42556.66 |
| miR-283-x | 23 | AAATATCAGCTGGTAATTCTGGG | 35225.92 | 51130.78 | 39083.04 | 44321.82 |
| miR-12-z | 22 | TGAGTATTACATCAGGTACTGG | 40292.51 | 44322.48 | 44485.41 | 39246.70 |
| miR-31-x | 23 | AGGCAAGATGTCGGCATAGCTGA | 41298.44 | 37804.15 | 40554.64 | 32662.48 |
| miR-9-z | 23 | TCTTTGGTTATCTAGCTGTATGA | 34828.74 | 31625.97 | 31174.85 | 24968.57 |
| miR-281-y | 22 | TGTCATGGAGTTGCTCTCTTTG | 32299.66 | 27567.92 | 31024.93 | 25282.89 |
| miR-306-x | 22 | TCAGGTACTGAGTGACTCTGAG | 28378.45 | 25029.68 | 26925.52 | 23335.59 |
| miR-279-y | 22 | TGACTAGAGTCACACTCGTCCA | 29215.03 | 26918.64 | 24475.01 | 21888.72 |
| miR-8-y | 23 | TAATACTGTCAGGTAAAGATGTC | 25944.71 | 24584.53 | 26698.57 | 24421.11 |
| miR-14-y | 22 | TCAGTCTTTTTCTCTCTCCTAT | 25756.03 | 24426.78 | 21402.83 | 18446.77 |
| miR-2-y | 23 | TATCACAGCCAGCTTTGATGAGA | 21556.81 | 21971.78 | 22704.16 | 22735.29 |
| miR-317-y | 21 | TGAACACAGCTGGTGGTATCT | 17680.66 | 14380.16 | 19697.04 | 16329.63 |
| let-7-x | 21 | TGAGGTAGTAGGTTGTATAGT | 16956.52 | 16026.12 | 15069.27 | 15996.02 |
| miR-375-y | 22 | TTTGTTCGTTCGGCTCGAGTTA | 13647.57 | 15387.23 | 10351.16 | 10257.95 |
| miR-13-y | 23 | TATCACAGCCATTTTGATGAGCT | 11571.52 | 11203.48 | 11346.38 | 10794.22 |
