# Supplementary material for: Comparative Identification of MicroRNAs in Apis cerana cerana Workers’ Midguts in Response to Nosema ceranae Invasion
Source: Insects. 2019 Aug 21;10(9):258. doi: 10.3390/insects10090258 (PMC6780218; doi:10.3390/insects10090258)
Supplement: Supplementary file 1 [file insects-10-00258-s001.zip › Supplementary Files/Table S5.doc]

Table S5. The expressed novel miRNAs in *A. c. cerana* worker's midgut.

| **miRNA** | **Length** | **Sequence** | **AcCK1**  **TPM** | **AcT1**  **TPM** | **AcCK2**  **TPM** | **AcT2**  **TPM** |
| --- | --- | --- | --- | --- | --- | --- |
| novel-m0019-3p | 21 | ACATCTACGATCGAGACGTAT | 239.01 | 225.22 | 277.17 | 227.50 |
| novel-m0001-3p | 22 | TCATTTCCGATTATTACCGAGT | 20.60 | 19.61 | 21.55 | 19.32 |
| novel-m0012-5p | 22 | ATGCACAGTTTGATCAACCGTT | 13.66 | 13.39 | 7.24 | 16.47 |
| novel-m0004-3p | 21 | TCTCTCGGCTCTTTGCCTGCT | 9.85 | 14.63 | 11.40 | 12.57 |
| novel-m0012-3p | 21 | TCGGTTTTAAAGTCTGTGCTA | 15.25 | 8.44 | 11.81 | 8.96 |
| novel-m0017-3p | 21 | TTTCGCGATTTTTTACGAGAT | 8.88 | 9.56 | 3.49 | 5.30 |
| novel-m0016-5p | 22 | ACGGAAAGGAGTTGGACGGACT | 4.56 | 6.80 | 9.79 | 4.85 |
| novel-m0010-5p | 23 | AAATTTCATTAGATGTCTGAATT | 6.38 | 4.54 | 9.33 | 1.62 |
| novel-m0009-5p | 22 | TAGAATATATAATTATGTCATT | 7.89 | 3.06 | 4.37 | 2.43 |
| novel-m0019-5p | 21 | AGTCTCGATCGAGACATGTGA | 6.46 | 1.22 | 8.37 | 0.01 |
| novel-m0003-3p | 24 | TGGTGATATGTGTATATACTGATT | 4.05 | 2.95 | 8.92 | 0.01 |
| novel-m0021-5p | 21 | ACTCGTGCGCAGCGACCGCGA | 3.91 | 4.80 | 2.26 | 3.75 |
| novel-m0020-5p | 24 | TTGTCTGAATACCCATTGGATCGT | 4.36 | 2.35 | 1.54 | 2.81 |
| novel-m0002-3p | 22 | TAAACATCGAGAGATAAGGATT | 5.15 | 2.04 | 1.09 | 2.50 |
| novel-m0006-3p | 22 | TATATATACACATACACACGTA | 3.30 | 0.01 | 1.90 | 4.11 |
| novel-m0022-3p | 21 | TCTCTCTGTCTCGCTTCGTCA | 0.56 | 3.78 | 2.52 | 1.62 |
| novel-m0007-3p | 22 | AATTTGACGTCATTTCTTGGAA | 3.40 | 1.22 | 0.73 | 2.43 |
| novel-m0015-3p | 22 | ATATCGACGAGAATAAATCGGC | 1.99 | 0.01 | 5.50 | 0.01 |
| novel-m0011-3p | 22 | CACTCTCACTTGTTTGGTGGGA | 1.30 | 2.16 | 2.31 | 1.62 |
| novel-m0008-3p | 22 | CTCAATCCGTGTTGAGACATTT | 6.03 | 0.92 | 0.01 | 0.01 |
| novel-m0023-5p | 22 | ACAGTCCCTGTGTTGTTCATGT | 2.54 | 3.06 | 0.83 | 0.01 |
| novel-m0005-3p | 22 | TCTCTTCTCGTGTCGCGAGAGA | 4.04 | 0.61 | 1.54 | 0.01 |
| novel-m0013-3p | 23 | TATTGTATCGTTAAAAGTATAAT | 2.75 | 1.83 | 0.83 | 0.01 |
| novel-m0014-3p | 23 | CCGGCGATGATGTCATGCAGTCT | 3.27 | 0.61 | 0.83 | 0.01 |
| novel-m0018-3p | 22 | AAGTCCCGGCACACCTGGAACT | 2.23 | 0.01 | 0.01 | 1.62 |
