# Supplementary material for: Comparative Identification of MicroRNAs in Apis cerana cerana Workers’ Midguts in Response to Nosema ceranae Invasion
Source: Insects. 2019 Aug 21;10(9):258. doi: 10.3390/insects10090258 (PMC6780218; doi:10.3390/insects10090258)
Supplement: Supplementary file 1 [file insects-10-00258-s001.zip › Supplementary Files/Table S6.doc]

**Table S6. Summary of DEmiRNAs in AcCK1 vs AcT1 comparison group.**

| **miRNA** | **Sequence** | **Length** | **TPM of miRNAs**  **in AmCK1** | **TPM of miRNAs in AmT1** | **Log_2_FC** | ***P* value** | **Mark** |
| --- | --- | --- | --- | --- | --- | --- | --- |
| miR-676-y | GCTGTCCTAAGGTAGATGA | 19 | 0.01 | 80.42 | 12.97 | 2.96E-05 | Up |
| miR-60-y | ACATGTTCTGGTTGAAGA | 18 | 0.01 | 18.70 | 10.87 | 1.51E-05 | Up |
| miR-2965-y | AGGACTGCTACAGAGAGCA | 19 | 0.01 | 17.17 | 10.75 | 3.91E-05 | Up |
| miR-8462-x | ATTAATTTGATAAGTTATA | 19 | 0.01 | 14.42 | 10.49 | 0.000242 | Up |
| miR-6717-x | GCGATGTGGGGACGGAGA | 18 | 0.01 | 12.06 | 10.24 | 2.14E-07 | Up |
| miR-6313-y | TGCTGTGAAGTTTTGATT | 18 | 0.01 | 5.97 | 9.22 | 2.21E-06 | Up |
| miR-3726-x | GAGTGGTGGATGCCAGCGTT | 20 | 0.01 | 5.17 | 9.02 | 0.000162 | Up |
| miR-252-y | CTGCTGCTCAAGTGCTTATCA | 21 | 11.17 | 27.02 | 1.27 | 0.025931 | Up |
| miR-980-y | AAGCTGCCTTTTGAAGGGCAACA | 23 | 39.09 | 18.85 | -1.05 | 0.038812 | Down |
| miR-598-y | GTCGTCGTCGTCATCGTCA | 19 | 2.41 | 0.01 | -7.92 | 9.16E-05 | Down |
| miR-1-x | CCGTGCTTCCTTACTTCCCATA | 22 | 3.34 | 0.01 | -8.38 | 2.82E-07 | Down |
| miR-965-x | GGGGAAAGGTTATAGCGATTATG | 23 | 3.64 | 0.01 | -8.51 | 4.12E-07 | Down |
| miR-4635-y | GAAGTCGGAACCCGCTAAG | 19 | 5.68 | 0.01 | -9.15 | 4.11E-05 | Down |
| miR-9204-x | CTGGGATGAAATGTGGGT | 18 | 6.02 | 0.01 | -9.23 | 0.00087 | Down |
