# Supplementary material for: Comparative Identification of MicroRNAs in Apis cerana cerana Workers’ Midguts in Response to Nosema ceranae Invasion
Source: Insects. 2019 Aug 21;10(9):258. doi: 10.3390/insects10090258 (PMC6780218; doi:10.3390/insects10090258)
Supplement: Supplementary file 1 [file insects-10-00258-s001.zip › Supplementary Files/Table S7.doc]

Table S7. Summary of DEmiRNAs in AcCK2 vs AcT2 comparison group.

| **miRNA** | **Sequence** | **Length** | **TPM of miRNAs**  **in AmCK2** | **TPM of miRNAs in AmT2** | **Log_2_FC** | ***P* value** | **Mark** |
| --- | --- | --- | --- | --- | --- | --- | --- |
| miR-676-y | GCTGTCCTAAGGTAGATGA | 19 | 0.01 | 410.36 | 15.32 | 4.02E-06 | Up |
| miR-60-y | ACATGTTCTGGTTGAAGA | 18 | 0.01 | 205.03 | 14.32 | 0.000287 | Up |
| miR-194-y | CAGTGGGGCGGTTGTTAT | 18 | 0.01 | 67.96 | 12.73 | 6.20E-06 | Up |
| miR-6313-y | TGCTGTGAAGTTTTGATT | 18 | 0.01 | 34.23 | 11.74 | 0.000177 | Up |
| miR-2965-y | AGGACTGCTACAGAGAGCA | 19 | 0.01 | 29.17 | 11.51 | 1.18E-06 | Up |
| miR-8462-x | ATTAATTTGATAAGTTATA | 19 | 0.01 | 18.29 | 10.84 | 4.28E-05 | Up |
| miR-7338-y | TTTAGCTGGTTTGTCAAGA | 19 | 0.01 | 9.56 | 9.90 | 2.31E-05 | Up |
| miR-3654-y | GCGACTGGAAAAGCTGAA | 18 | 0.01 | 6.91 | 9.43 | 2.64E-06 | Up |
| miR-3720-x | TACGGTGATGAGTTTAAA | 18 | 24.49 | 63.42 | 1.37 | 0.023317 | Up |
| novel-m0019-5p | AGTCTCGATCGAGACATGTGA | 21 | 8.37 | 0.01 | -9.71 | 1.58E-06 | Down |
| novel-m0003-3p | TGGTGATATGTGTATATACTGATT | 24 | 8.92 | 0.01 | -9.80 | 8.91E-05 | Down |
| miR-92-x | TTGGGCGGGGTGTCCGTGC | 19 | 31.95 | 0.01 | -11.64 | 0.001613 | Down |
